# Effective Social Relationship Measurement and Cluster Based Routing in Mobile Opportunistic Networks †

**DOI:** 10.3390/s17051109

**Published:** 2017-05-12

**Authors:** Feng Zeng, Nan Zhao, Wenjia Li

**Affiliations:** 1 School of Software, Central South University, Changsha 410083, China; nanzhao@csu.edu.cn; 2 Department of Computer Science, New York Institute of Technology, New York, NY 10023, USA

**Keywords:** mobile opportunistic network, routing, clustering, social relationship, circles of friends

## Abstract

In mobile opportunistic networks, the social relationship among nodes has an important impact on data transmission efficiency. Motivated by the strong share ability of “circles of friends” in communication networks such as Facebook, Twitter, Wechat and so on, we take a real-life example to show that social relationships among nodes consist of explicit and implicit parts. The explicit part comes from direct contact among nodes, and the implicit part can be measured through the “circles of friends”. We present the definitions of explicit and implicit social relationships between two nodes, adaptive weights of explicit and implicit parts are given according to the contact feature of nodes, and the distributed mechanism is designed to construct the “circles of friends” of nodes, which is used for the calculation of the implicit part of social relationship between nodes. Based on effective measurement of social relationships, we propose a social-based clustering and routing scheme, in which each node selects the nodes with close social relationships to form a local cluster, and the self-control method is used to keep all cluster members always having close relationships with each other. A cluster-based message forwarding mechanism is designed for opportunistic routing, in which each node only forwards the copy of the message to nodes with the destination node as a member of the local cluster. Simulation results show that the proposed social-based clustering and routing outperforms the other classic routing algorithms.

## 1. Introduction

Mobile Opportunistic Networks (MONs) can be formed by wireless portable devices such as iPads, PDAs, smartphones, etc., which are usually carried around by human beings. Due to the random mobility of nodes, there are no persistent connections between any two nodes. For data transmission, each node stores data to be sent and then forwards them to the encounter nodes. Such a data delivery process refers to the “storage-carry-and-forward” mechanism, which is the basic principle for data transmission and routing in MONs. Since the path from a source to a destination is intermittently connected, the conventional routing protocols are generally not applicable, and routing becomes a challenging issue in MONs [1].

Based on the “storage-carry-and-forward” data transmission mode, two simple algorithms were proposed in the research on opportunistic routing. One is Epidemic Routing [2], and the other is Direct Transmission [3]. In Epidemic Routing, each node simply forwards data to all encountering nodes. Obviously, Epidemic Routing has the highest data delivery success rate among all routing algorithms but also has the highest network overhead. On the contrary, in Direct Transmission, the source node stores the data to be sent and does not forward them to any nodes until it reaches the destination. Direct Transmission had the lowest network overhead but also the lowest data delivery success rate. The other research works on routing in MONs tried to create a possible tradeoff between data delivery rate and network overhead.

As human beings take part in network activities, the behavior of mobile nodes thus shows certain social attributes. Some research works [4] had shown that social relationships among nodes had an important impact on node encounter events and their duration time, which would be useful for reducing routing overhead improving the success rate of data transmission. Accordingly, a number of researchers made good use of nodes’ social attributes to design data forwarding mechanisms and received good results. In the long run, the data transfer mechanism based on social relationships among nodes will be more stable than the other forwarding modes [4].

In social based routing, the key point is to find and measure the social relationships among nodes, which depend on the analysis of the historical data of encounters of nodes [5,6]. Some works used historical data to predict the probability of the nodes’ encounters, and thereby designed optimal algorithms for message forwarding. Some works measured strength of social relationships among nodes based on nodes’ encounter frequency, formed social clusters of nodes, and proposed cluster-based routing mechanism for message forwarding. However, as for social relationship finding and measurement, there are still some important points not considered in the existing research work. For example, there is such a social phenomenon in the real world: Bob has two grandmothers represented by *D* and *E*, *D* is the mother of Bob’s mother, and *E* is the mother of Bob’s father. Although *D* and *E* are close relatives, they may never contact each other because of their opposing personalities. In this phenomenon, with *D* and *E* acting as two nodes in MONs, they have no direct encounter and link, but, in fact, there is a strong social relationship between them. Therefore, in some conditions, we may fail to find and measure the social relationship between two nodes only from their direct encounter records.

In fact, although *D* and *E* have no direct relationship, they have common close friends including Bob and his parents. Observing their circles of friends, we can find out how close the social relationship between *D* and *E* is. Consequently, the social relationship between two nodes can be divided into two parts. One part comes from direct contact events, which are called Explicit Social Relationships in this paper. Another part is called Implicit Social Relationships obtained from the common friends of the two nodes. Like the communication networks such as Facebook, Twitter, Wechat and so on, the circles of friends can be used to find the relationship between two people.

In this paper, we study social relationships among nodes, and propose social based clustering and routing mechanisms in WONs. Inspired by some social phenomena, we present the definitions of explicit and implicit social relationships, which are used to determine the strength of social relationships among nodes. Our experiment results show that the proposed methods can exactly evaluate social relationships among nodes, which is helpful for routing mechanisms to improve data delivery rates. The contributions of this paper are listed as follows:

- We present the definitions of explicit social relationships (ExSRs) and implicit social relationship (ImSRs), and combine both ExSRs and ImSRs to measure social relationships between nodes. Adaptive weights are given to ExSRs and ImSRs in the measurement of social relationships, which can be adjusted to the contact feature of the nodes, and thus accuracy measurement of social relationship will be achieved. In addition, the distributed computing scheme of common friends is proposed for the calculation of ImSR.
- We propose a novel social-based clustering and routing scheme. Each node selects the nodes with closed social relationships to form a local cluster, and the self-control method is used to keep all cluster members always having close relationships with each other. A cluster-based message forwarding mechanism is designed for opportunistic routing, in which each node only forwards the copy of the message to nodes with the destination node as a member of local cluster.

The rest of this paper is structured as follows. In Section 2, we describe and analyze the related work. In Section 3, definitions and computing methods are presented for explicit and implicit social relationships. In Section 4, social-based clustering and routing is presented and analyzed. Simulation results are presented in Section 5. The last section concludes this paper.

## 2. Related Work

Opportunistic routing has been extensively studied, and various types of message-forwarding methods and routing algorithms were proposed for “storage-carry-and-forward” data transmission with the goal of reducing network overhead and improving data delivery rate. Epidemic Routing [2] flooded the message to all nodes without consideration of routing overhead. Theoretically, Epidemic Routing had the highest success rate of data delivery, but at the price of highest routing overhead. Direct Transmission [3] required that the source stored the data to be sent, and did not forward the data to any nodes but the destination. Direct Transmission had the lowest overhead among all routing algorithms, but also had the lowest success rate of data delivery. Other works in opportunistic routing tried to make a balance between Epidemic Routing and Direct Transmission, and create a possible tradeoff between data delivery rate and routing overhead. Lindgren et al. [7] used historical data of nodes’ encounters and transmission records to compute forwarding probabilities between nodes and the proposed forwarding-probability based routing mechanism PRoPHET, which had the approximate success rate of data delivery of Epidemic Routing [2] with the low routing overhead. Spyropoulos et al. [8] took the advantages of Epidemic Routing and Direct Transmission into consideration and proposed a routing scheme called Spray and Wait, which “sprayed” a number of copies into the network, and then “waited” until one of these nodes met the destination. Erramilli et al. [9] proposed a delegation forwarding solution for opportunistic routing, which required a node to forward data to only the node with the highest forwarding performance so far. Balasubramanian et al. [10] treated opportunistic routing as a resource allocation problem, and each node replicated packets according to packet utility value, which was determined by the delay to destination and encounter probability with destination. Zhang et al. [11] proposed a novel mobility prediction-based routing, and the computation of the probability for a node destined to an area was based on the semi-Markov model. In [12,13,14], the authors used network coding in opportunistic routing to improve throughput. The above routing schemes used a variety of mechanisms, including discovering the meeting probabilities among nodes, packet utility, and network coding. The primary focus of these mechanisms was to increase the likelihood of finding a path with limited information, and the effectiveness had a relationship with the accuracy of prediction models or evaluation methods.

As human beings take part in network activities, the behavior of mobile nodes therefore shows some certain social attributes. Research work [4] had shown that social relationships among nodes could be used to improve the performance of opportunistic routing. Recently, there have been some interesting works based on social relationships among nodes. Hui et al. [15] proposed a social-based routing named BUBBLE. The authors exploited two social and structural metrics, namely, centrality and community. Based on the social activity of nodes, they calculated the global ranking of the nodes in the whole network and the local ranking of the nodes in the community. The message was forwarded to the nodes with high social activity ranking. Gao et al. [16] studied multicast in Delay Tolerant Networks (DTNs) from the social network perspective, used the cumulative probability of node encounter to get node centrality and social community structures, and selected the nodes with high node centrality as the relay nodes in multicast. Fan et al. [17] explored the relationship between geographic and social regularities of human mobility, proposed the concepts of geocommunity and geocentrality, used the semi-Markov process to model user mobility based on the geocommunity structure of the network, and proposed route algorithms to minimize total duration or maximize the dissemination ratio. Wei et al. [18] took the use of frequency and duration of node contacts to generate the social graph, addressed the community evolution problem, proposed distributed algorithms based on the social graph to detect the overlapping communities and bridge nodes, and designed a social-based routing scheme. Orlinski et al. [19] studied cluster based routing, and proposed a routing scheme with cluster size adjusted dynamically. Mtibaa et al. [20] developed the PeopleRank approach for node ranking, which was similar to PageRank. PeopleRank gave higher weight to nodes that were socially connected to other important nodes of the network. In message forwarding, a node *u* forwarded a message to a node *v* that it encountered if the rank of *v* was higher than the rank of *u*. Mei et al. [21] proposed a socially aware and stateless routing scheme called SANE, in which message forwarding was based on the interest similarity between nodes. SANE had the advantage of requiring less of a buffer of nodes than other approaches. However, the interest is just one of the social ties between nodes. For the nodes having no common interest, but frequent contact with the destination, they can often make a great contribution to message delivery, as is not considered in SANE.

From different viewpoints of social relationships among nodes, the above works proposed the effective routing solutions for a variety of problems, which had shown that social relationships among nodes had a major impact on the performance of opportunistic routing. With the growing popularity of smartphones, more and more people have the opportunity to participate in a variety of applications of MONs, and comprehensive study of the social relationships among nodes will be more important than before in MONs.

Different to the existing research work, in this paper, we take a real social phenomenon as an example to discuss the measurement methods of social relationships, and propose a novel social-based scheme for clustering and routing in MONs. In our daily lives, we can get a lot of information from the circles of friends in social communication networks such as Facebook, Twitter, Wechat, and so on, which inspires us to analyze the social relationships among nodes in MONs. In the real world, the social relationship between two people is not only shown in direct contact with each other, but also in their common circles of friends. For example, in our lives, there is often such a phenomenon: Bob has two grandmothers *D* and *E*: *D* is the mother of Bob’s mother, and *E* is the mother of Bob’s father. Although *D* and *E* are close relatives, they may never contact each other because of their opposing personalities. In this phenomenon, there is no direct contact between *D* and *E*, but, in fact, they have strong social relationships because they have a common “circles of friends”. As the saying goes, “like attracts like”, and we have many types of circles of friends such as working circles, classmate circles, common hobby circles and so on. Real-life experience tells us that we can get important information from circles of our friends.

In an MON, the nodes are often wireless devices carried by human beings, and thus the activities of nodes are inevitable for showing some social characteristics described above. Consequently, the study of social relationships should reveal the explicit relationships among nodes via direct contact events, and also determine the implicit social relationships among them from their circles of friends. In this paper, we divide the social relationships between two nodes into two parts. One part comes from direct contact events, which are called Explicit Social Relationships (ExSRs). Another part of relationships should be relayed by their common friends, called Implicit Social Relationships (ImSRs). We present the measure methods of ExSRs and ImSRs, which are used in the design of clustering and routing. In short, what we focus on in this paper is measuring implicit social relationships between nodes from their circles of friends, and the accurate measurement of social relationships can improve the performance of the social-based routing proposed in this paper.

## 3. Social Relationship Measurement

As discussed above, the social relationships between nodes includes two parts: explicit social relationships and implicit social relationships. The social relationships between nodes can be represented by the combination of these two parts.

### 3.1. Definitions

It is supposed that node *i* encounters node *j* at time *t*, and node *i* can measure its social relationship $S_{ij}\left(t\right)$ with node *j*. $S_{ij}\left(t\right)$ is the strength of the social relationship from node *i* to *j* at time *t*, and $E_{ij}\left(t\right)$ and $H_{ij}\left(t\right)$ are explicit and implicit parts of $S_{ij}\left(t\right)$, respectively. Then, $S_{ij}\left(t\right)$ can be denoted as Equation (1), where $W_{i}^{E}\left(t\right)\in \left[0,1\right]$ and $W_{i}^{H}\left(t\right)\in \left[0,1\right]$ are used to adjust the weights of explicit and implicit social relationships for the strength of social relationships with $W_{i}^{E}\left(t\right)+W_{i}^{H}\left(t\right)=1$. Depending on the frequency of node encounters, the weight functions $W_{i}^{E}\left(t\right)$ and $W_{i}^{H}\left(t\right)$ may vary over time:

(1) $$S_{ij}\left(t\right)=W_{i}^{E}\left(t\right)E_{ij}\left(t\right)+W_{i}^{H}\left(t\right)H_{ij}\left(t\right).$$

In MONs, we suppose that each node has an encounter table, which stores node identification and encounter frequency during recent time *T*. $N_{i}\left(t\right)$ is the set of nodes encountered by node *i* during recent time *T* at time *t*, and $N_{ij}\left(t\right)=N_{i}\left(t\right)\cap N_{j}\left(t\right)$ is the set of nodes encountered by both nodes *i* and *j*. We also say that $N_{ij}\left(t\right)$ is the set of common friends of nodes *i* and *j* at time *t*, or the circles of friends of node *i* and *j*. In addition, $f_{ij}\left(t\right)$ is the number of encounter times between node *i* and *j* during recent time *T* at time *t*. Due to the symmetry of node encounters, we have $N_{ij}\left(t\right)=N_{ji}\left(t\right)$ and $f_{ij}\left(t\right)=f_{ji}\left(t\right)$.

Observing social phenomena in our real lives, we find that if nodes *i* and *j* have social relationships with each other, they must meet one of the following two conditions: (1) they have direct contact with each other, and (2) they have a common circle of friends. In this paper, the two conditions above form Explicit Social Relationships (ExSRs) and Implicit Social Relationships (ImSRs), respectively.

**Definition** **1** (Strength of ExSR). *The strength of ExSR ($E_{ij}\left(t\right)$) between nodes i and j at time t is denoted as Equation (2), where μ is a constant integer and the threshold of encounter times. If the encounter times between nodes i and j is more than μ, the strength of ExSR will be equal to* 1*, which means a close ExSR between node i and j. In general, μ is an integer, set as 1.2 times the average encounters among all nodes. As shown in Equation (2), $E_{ij}\left(t\right)\in \left[0,1\right]$ is determined by encounter times of nodes i and j:*

(2) $$E_{ij}\left(t\right)=\frac{min(\mu ,f_{ij}\left(t\right))}{\mu }.$$

**Definition** **2** (Strength of ImSR). *The strength of ImSR ($H_{ij}\left(t\right)$) between nodes i and j at time t is denoted as Equations (3) and (4), where $R_{ij}\left(t\right)$ is used to denote the encounter times between nodes i or j and their common circles of friends, and represents the indirect message delivery capability between nodes i and j. Equation (3) has the same μ as Equation (2). $H_{ij}\left(t\right)\in \left[0,1\right]$ depends on the common circles of friends between node i and j, and the bigger size and more frequent contact of common circles of friends will lead to a stronger ImSR with each other:*

(3) $$H_{ij}\left(t\right)=\frac{min(\mu ,R_{ij}\left(t\right))}{\mu },$$

(4) $$R_{ij}\left(t\right)=\sum_{k\in N_{ij}\left(t\right)}min\left(f_{ik}\left(t\right),f_{jk}\left(t\right)\right).$$

**Figure 1:**
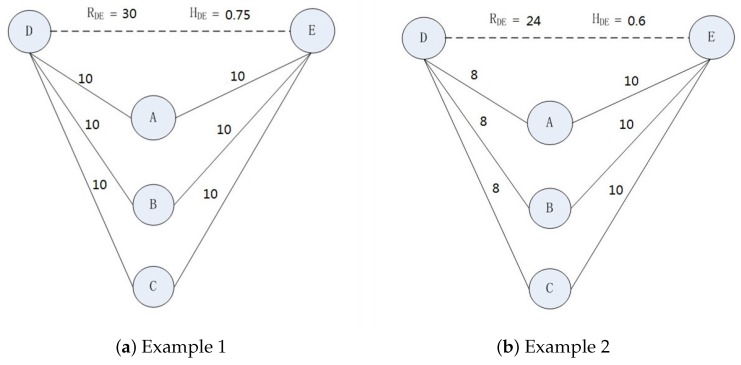
Implicit Social Relationships.

As shown in Figure 1, nodes *A*, *B* and *C* stand for Bob and his parents, respectively, as is mentioned in Section 1, *D* is the mother of Bob’s mother, and *E* is the mother of Bob’s father. The encounter times between two nodes are marked with the solid lines. Supposing μ=40, according to Equations (3) and (4), we can get $R_{DE}=min\left(10,10\right)+min\left(10,10\right)+min\left(10,10\right)=30$ and $H_{DE}=\frac{30}{40}=0.75$ in Figure 1a, and $R_{DE}=min\left(8,10\right)+min\left(8,10\right)+min\left(8,10\right)=24$ and $H_{DE}=\frac{24}{40}=0.6$ in Figure 1b. Since *D* and *E* do not have direct contact at all, the communication between *D* and *E* should be relayed by *A*, *B* or *C*. Therefore, if *A*, *B* and *C* reduce the contact with *D* or *E*, the message delivery capability between *D* and *E* will have a certain degree of decline. In Figure 1a,b, $R_{DE}$ is 30 and 24, respectively, which reflects the message delivery capability between *D* and *E*. Since the contact strength between {A,B,C} and *D* in Figure 1a is more than that in Figure 1b, the message delivery capability between *D* and *E* in Figure 1a will be better than that in Figure 1b.

### 3.2. Adaptive Weights of ExSR and ImSR

**Figure 2:**
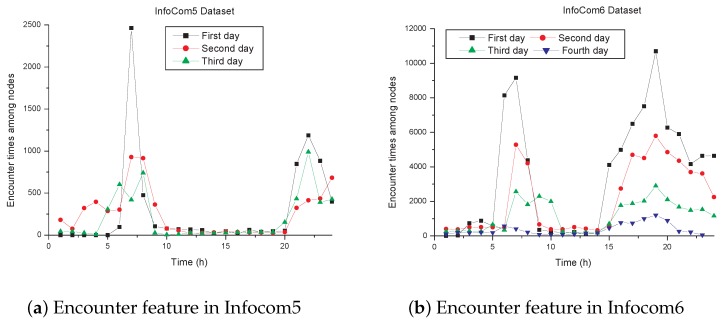
Encounter feature in datasets of Infocom5 and Infocom6.

From analysis of the real datasets Infocom5 and Infocom6, we find that the number of node encounters changes greatly in different time periods, as is shown in Figure 2. From 7:00 a.m. to 9:00 a.m., and 6:00 p.m. to 10:00 p.m., the nodes have high frequency of encounter. However, the frequency of node encounter is low from 10:00 a.m. to 5:00 p.m., and 11:00 p.m. to 5:00 a.m. next day. This feature is consistent with our real lives. Our commuting time is from 7:00 a.m. to 9:00 a.m., and 6:00 p.m. to 10:00 p.m., and the large flow of people increases the encounter number between individuals. However, during the rest of the daytime, we may always stay with families, friends, and co-workers, and thus our encounter number with others goes down.

From the above analysis, we can find that there are two parts of time each day, i.e., commuting time and resting time, and two corresponding features of encounter events among people in our daily lives. One feature is that we encounter a lot of people during our commuting time. Because the encounter time is short, and most people are strangers to us, this means that there are almost no common friends between us and the people we meet. The other feature is that we are relatively static during the rest of the daytime, staying with people who we are familiar with, which means that, in this part of the time, we usually have common friend circles with the people we encounter, and the encounters during this time are relatively long. As is shown in the above analysis, ExSRs put emphasis on direct contact between nodes, and ImSRs are related with common friend circles. Consequently, when most of the relationships between nodes are direct contact, we want increase to the weight of ExSRs in social relationships among nodes. With direct contact decreasing or common friends increasing, ImSRs should have increasing weight in social relationships among nodes. In order to measure the frequency of direct contact among nodes on the whole, we define the average contact frequency around node *i* at time *t* as $f_{i}\left(t\right)$ shown in Equation (5):

(5) $$f_{i}\left(t\right)=\frac{\sum_{j\in N_{i}\left(t\right)}f_{ij}\left(t\right)+\sum_{j\in N_{i}\left(t\right)}\sum_{k\in N_{j}\left(t\right)}f_{jk}\left(t\right)}{|N_{i}\left(t\right)|+1}.$$

Then, the weights of ExSRs and ImSRs in social relationships from node *i* to other nodes are defined in Equations (6) and (7), respectively. In Equation (6), μ is the same as Equation (2):

(6) $$W_{i}^{E}\left(t\right)=\frac{min(f_{i}\left(t\right),\;\mu )}{\mu },$$

(7) $$W_{i}^{H}\left(t\right)=1-W_{i}^{E}\left(t\right).$$

### 3.3. Distributed Computing for Social Relationships

In MONs, each node has an encounter table to record encounter events. Consequently, based on Equation (2), each node can use its own encounter table to calculate the ExSR with other nodes.

When two nodes encounter each other, they will exchange their encounter table. With the exchange of encounter tables, the ExSR between two nodes can be calculated based on the information of their direct contacts, and each node can update its circles of friends with other nodes based on the following distributed computing method. In order to collect common friends with other nodes, each node maintains a common friend table. According to the information in the common friend table, each node can calculate the ImSRs with other nodes. With the Figure 1b as an example, the distributed computing method for ImSRs can be described as follows.

**Figure 3:**
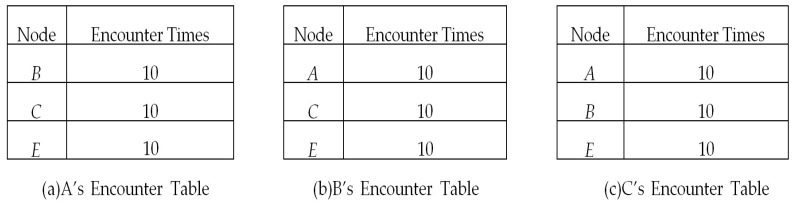
Encounter tables of nodes *A*, *B* and *C*.

It is supposed that nodes *A*, *B* and *C* have the encounter tables shown in Figure 3, which are used for information exchange with *D*; thus, *D*’s information is removed from these tables. Then, nodes *A*, *B* and *C* are going to encounter node *D* one by one.

**Figure 4:**
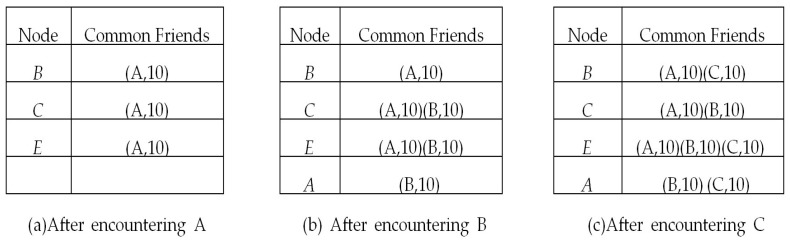
D’s common friends with other nodes.

As mentioned above, each node has a common friend table storing the information of common friends with other nodes. When node *A* encountered *D*, they exchanged the encounter tables with each other, and node *D* got the encounter table of *A*, which is shown in Figure 3a. Because *D* and *A* have encountered each other, *A* becomes one of *D*’s friends. From *A*’s encounter table, based on the symmetry of encounter events, *A* is the friend of *B*, *C* and *E*, and nodes *B*, *C* and *E* have 10, 10, and 10 encounters with node *A*, respectively. Consequently, we can deduce that node *A* is one of the common friends of nodes *D*, *B*, *C* and *E*. Therefore, after an encounter with node *A*, node *D* has the common friend table shown in Figure 4a. The first tuple (*A*, 10) in Figure 4a means that nodes *D* and *B* have a common friend *A*, and the value 10 denotes the encounter times between *B* and *A*. It should be mentioned that the encounter times between *D* and *A* can be obtained from *D*’s encounter table. Similarly, after encountering *B* and *C*, node *D* had the common friend table shown in Figure 4b,c respectively.

As shown in Figure 4c, after encountering *A*, *B* and *C*, *D* finds that it has *A*, *B* and *C* as common friends with *E*, and *E* has encounters 10 times with each of *A*, *B* and *C*, which are marked as (*A*, 10), (*B*, 10) and (*C*, 10) in the table item of node *E*. From Figure 1b, *D* has encounters with each of *A*, *B* and *C* eight times. Based on Equations (1) to (7) with μ=40, the social relationship between *D* and *E* ($S_{DE}$) can be calculated as follows.

When node *D* encounters *A*, *B* and *C*, *D* can get their encounter tables as shown in Figure 3, and *D* can find that each of *A*, *B* and *C* has direct contact with other nodes 30 times. From Figure 1b (from its own encounter table), node *D* has direct contact with other nodes 24 times. Therefore, we can get $f_{D}=28.5$, and $W_{D}^{E}\left(t\right)=0.7125$, and

$$\begin{array}{l} f_{D}=\frac{24+30+30+30}{4}=28.5,\\ W_{D}^{E}\left(t\right)=\frac{min(28.5,40)}{40}=0.7125,\\ \begin{array}{cc} S_{DE} & =0.7125\times E_{DE}+\left(1-0.7125\right)\times H_{DE}\\ & =0.7125\times \frac{min(40,\;0)}{40}+0.2875\times \frac{min[40,min(8,\;10)+min(8,\;10)+min(8,\;10)]}{40}\\ & =0.7125\times 0+0.2875\times 0.6\\ & =0.1725. \end{array} \end{array}$$

The distributed exchange of node information may raise some privacy and security concerns. If the information is exchanged between nodes in plain text, the nodes can acquire their neighbors’ situations about the most encountered nodes, which are sometimes private for the related mobile device carriers. Moreover, the malicious users can falsify the exchanged information and appear to have a close relationship with the destinations, so that other nodes may wrongly forward the message to them. Therefore, the destinations may be attacked by malicious users by means of falsifying the original message. In this paper, we focus on social relationship measurement and the efficient routing schemes, which may have security and privacy problems. Recently, a number of solutions [22,23,24,25,26] have been proposed to deal with the security and privacy issues in information exchange between nodes in MONs. The solutions to the privacy and security problems in the proposed scheme can refer to the related works mentioned above.

## 4. Social-Based Clustering and Routing

With effective measurement of social relationships among nodes, we propose a Social-based Clustering and Routing scheme (SCR) in MONs, including the construction and update of clusters, and cluster-based routing.

### 4.1. Cluster Construction and Update

In the SCR scheme, each node has an encounter table recording the set of encounter nodes and corresponding encounter times during recent time *T*. Based on the encounter table, each node can calculate the ExSR with other nodes. In addition, each node has a common friend table recording its common friends with other nodes, which can be called circles of friends and is used to compute ImSR with other nodes. After social relationship measurement, each node selects the nodes with close social relationships to join its local cluster, which will be updated dynamically to control the size and keep the close relationship among members. For cluster updating, each node has a delete list marking the nodes going to be dropped from the local cluster. For node *i*, $N_{i}$, $C_{i}$ and $D_{i}$ are its sets of encounter nodes, local cluster and delete list, respectively.

Initially, the related tables and lists in each node are empty. When two nodes encounter each other, they update their encounter table and exchange encounter information. After information exchange, they update their common friend tables similar to the example shown in Figure 3 and Figure 4. Then, based on Equations (1) to (7), they calculate their strength of social relationship with other nodes. The encounter of two nodes may bring the change of their common friends with other nodes, thus their social relationship strength with other nodes may change.

When node *i* encounters node *j*, it is supposed that $S_{ij}\left(t\right)$ is the strength of the social relationship between nodes *i* and *j* at time *t*, and the parameter ω is the threshold of social relationship strength for cluster members. Cluster construction and update are discussed as follows according to different conditions of $S_{ij}\left(t\right)$ and ω.

**Condition 1: $S_{ij}\left(t\right)$ is greater than ω**

If $S_{ij}\left(t\right)$ is greater than ω, and node *j* is not a member of $C_{i}$, node *j* will join $C_{i}$. Otherwise, if node *j* is a member of $C_{i}$ and in the delete list $D_{i}$, node *j* will be removed from $D_{i}$, and node *j* is avoided being dropped out from the cluster.

**Condition 2: $S_{ij}\left(t\right)$ is equal to or less than ω**

If $S_{ij}\left(t\right)$ is equal to or less than ω, and node *j* is not a member of $C_{i}$, node *i* will do nothing for node *j*. Otherwise, if node *j* exists in $D_{i}$, node *j* will be removed from $C_{i}$ and $D_{i}$, which means that node *j* is dropped out from the local cluster of node *i*. However, if node *j* does not exist in $D_{i}$, node *j* will enter the delete list $D_{i}$ waiting for future consideration of removal from the local cluster.

**Figure 5:**
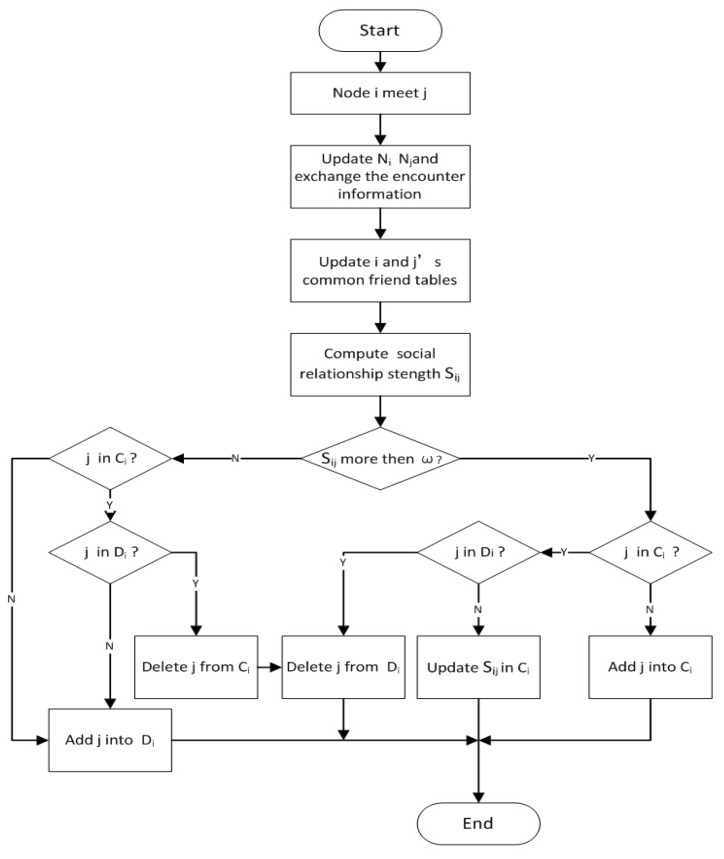
Cluster update.

The local cluster in each node will be updated dynamically, and the above processing will be done if the related information has a change. The processing of cluster update can be shown in Figure 5.

In every calculation of a social relationship between a node and its cluster members based on Equations (1) to (7), the result will be stored in the local cluster table. Before the next calculation, the stored strength of the social relationship will decrease a certain value over time, which can be helpful for dropping the out-of-date nodes from the local cluster, controlling the size of cluster, and keeping the close relationship between a node and its cluster members. It is mentioned above that the time *T* is the period for node encounter information collection, thus if no information shows that a node is alive, the node will enter an observing window for consideration of dropping out from the local cluster. It is supposed that there are *n* periods for the social relationship strengths to decrease to the threshold ω. Consequently, the social relationship strengths in cluster table will be self-decreased by a value ζ every $\frac{T}{n}$ time. To social relationship strength between node *i* and its cluster member *j*, the decreasing value in each period is $\zeta_{ij}$ shown in Equation (8). If node *j* is one of cluster members of node *i*, and the recorded social relationship strength $S_{ij}$ has not been updated for a period of time *T*, it will be self-decreased to ω. Then, node *j* will put onto the delete list of node *i*, and wait for consideration of dropping from the local cluster of node *i*:

(8) $$\zeta_{ij}=\frac{S_{ij}-\omega }{n}.$$

### 4.2. Cluster-Based Routing

As described above, each node selects the nodes with a close social relationship to form a local cluster. Making full use of the close relationship among cluster members, cluster-based routing can improve the success rate of data delivery, and also cut down on routing overhead. In this paper, when two nodes encounter each other, message forwarding happens only if the destination is in the local cluster of the encountering node. The process of routing can be described in two stages. One is information exchange and the other is message forwarding.

**(1) Information exchange**

When two nodes encounter each other, they send a “hello” message to each other, and then they get node identity and node encounter information of the other node. With the received information, they can update their encounter table and common friend table, which are used to calculate the strength of the social relationship with other nodes, and set up their local cluster.

**(2) Message forwarding**

It is supposed that node *i* encounters node *j*, and node *i* has the message to forward. If node *j* is the destination of the message, node *i* will forward the message to *j* and delete the message from its sending queue. Otherwise, there are the following two cases for node *i* to forward the message.

**Case 1: Intra-cluster message forwarding**

If the destination of the message is in the local cluster of node *i*, and node *j* is also a member of the cluster, node *i* will forward a copy of the message to node *j*. Otherwise, node *i* does not forward the message to node *j*.

**Case 2: Inter-cluster message forwarding**

If the destination of the message is not a member of the local cluster of node *i*, node *i* will send a “Request” packet to node *j* requesting whether the destination is in its local cluster. After node *j* receives the “Request” packet and checks the cluster table according to the request, node *j* will reply to node *i* with a “Response” packet notifying it about whether or not the destination is a cluster member of node *j*. If node *i* receives the “Response” packet and the destination is a cluster member of node *j*, node *i* will forward the message to node *j*. Otherwise, node *i* does not forward the message to node *j*.

The message to be sent can stay in the buffer of node *i* for a period of time that is dependent on the buffer size of node *i*. During this period of time, if node *i* encounters the forwarding objectives mentioned in the above rules, the message will be scheduled to be sent. Otherwise, the message will be dropped from the buffer of node *i* according to the buffer management algorithm. There exists a possibility that the message can not be forwarded if none of the nodes have destinations as local cluster members, and the large buffer size will be helpful for message delivery.

## 5. Simulations

We implement the proposed scheme SCR in the Opportunistic Network Environment simulator (ONE) [27] simulator, and evaluate SCR by performance comparison with PRoPHETv2 [7], DRAFT [19] and BUBBLE [15]. In simulation, the real datasets Infocom5, Infocom6, Cambridge and Intel are used for node activity driving, which can be downloaded from CRAWDAD [28]. The last updating date of the datasets was in August 2016, and the detailed information is shown in Table 1. In simulation, the node buffer size is set to 5M, the message size is 1K, and the node number and TTL are different to the four datasets shown in Table 2. In SCR, the parameters are set as ω=0.4, n=500, and μ as 1.2 times the average encounters among all nodes in each dataset. In DRAFT, the parameters are set as τ=7, δ=0.9 and t=3600 s.

We compare the performance of each routing algorithm in the same simulation environment and analyze the impact of parameters on SCR. The following metrics are used in the performance comparison.

**Packet Delivery Ratio (PDR):** The ratio of the number of data packets that successfully reached the destination and the amount of data packets sent by the source within a certain time.

**Transmission Delay (TD):** The delay is the average time it takes for a packet to reach the destination after it leaves the source.

**Routing Overhead Ratio (ROR):** As is shown in Equation (9), the total number of packets to be forwarded (relayed_number) minus the number of packets successfully transferred to the destination node (delivered_number), and then divided by the number of packets successfully transferred to the destination:

(9) $$Routing\_Overhead\_Ratio=\frac{relayed\_number-delivered\_number}{delivered\_number}.$$

### 5.1. Packet Delivery Ratio

**Figure 6:**
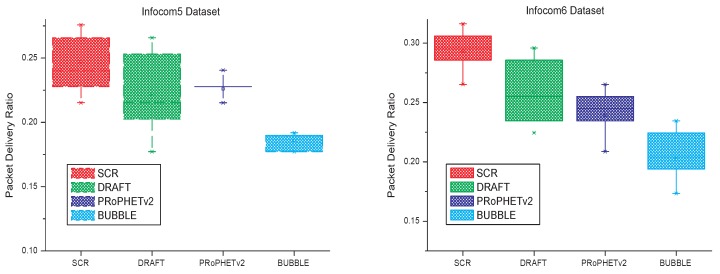

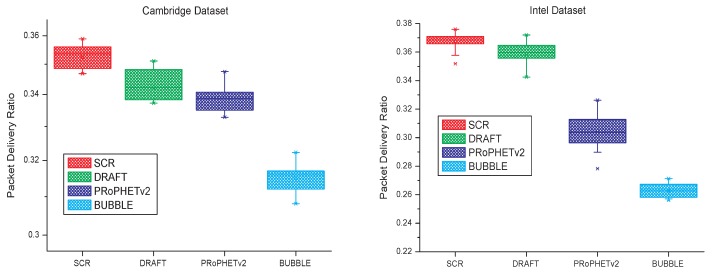
Quartiles of packet delivery ratios.

In the simulation, SCR, PRoPHETv2, DRAFT and BUBBLE run in the four datasets respectively, and the simulation time is the duration of dataset, which is shown in Table 1. Simulation results are shown in Figure 6. Similar to [19], quartiles are used to analyze the experimental results. The experimental data is arranged in ascending order and then divided into four equal parts. Then, we can find In Figure 6 there are five signs (min, first quartile, median, third quartile and max) for the result of each algorithm. In some special situations, two or more signs may have the same value. For example, in the first part of Figure 6, to the result of BUBBLE, the first quartile and median have the same value. The quartiles shown in Figure 6 can reflect the distribution center, concentration and spread range of packet delivery ratio. As can be seen from Figure 6, SCR has a higher distribution center of packet delivery ratio than the other three algorithms, a small spread range and a focus on better range.

**Figure 7:**
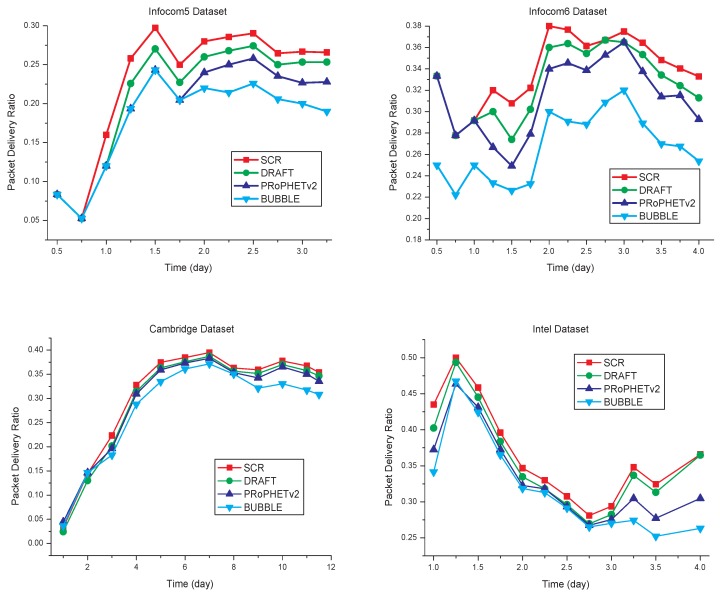
Packet delivery ratio comparisons.

With the simulation time varying, the results are shown in Figure 7. When simulation time is less than one day, SCR has similar results to the other algorithms. Due to the formation of clusters in the network requiring a process, in a short simulation time, there are not enough nodes in the local cluster for efficient data transmission. With the increasing of simulation time, SCR has a higher average packet delivery ratio than the other three algorithms. Compared with PRoPHETv2, DRAFT and BUBBLE, SCR has the higher packet delivery ratio, as shown in Table 3.

### 5.2. Transmission Delay

**Figure 8:**
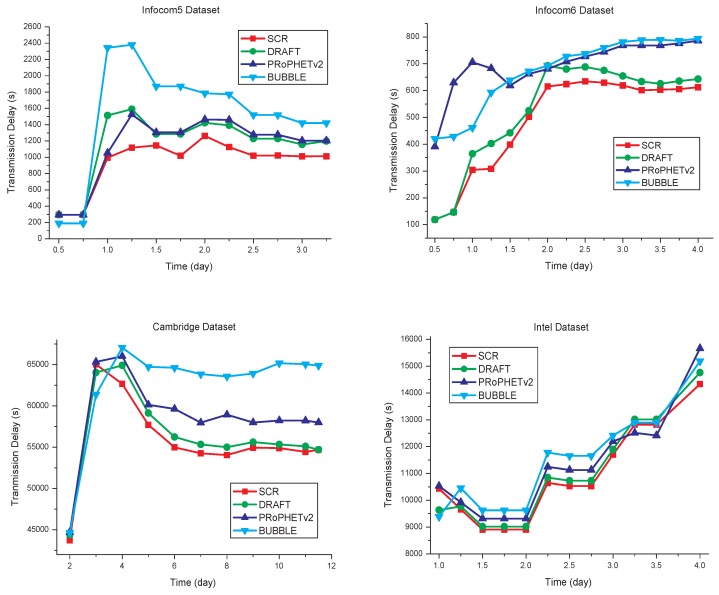
Transmission delay comparisons.

The transmission delay of each algorithm is shown in Figure 8. Compared with the other three algorithms, SCR has the transmission delay decreased by some extent, which is shown in Table 4. Since SCR is cluster-based routing, and cluster members have strong social relationships with each other, it can reduce unnecessary data transmission, and thereby reduce the transmission delay.

### 5.3. Routing Overhead Ratio

**Figure 9:**
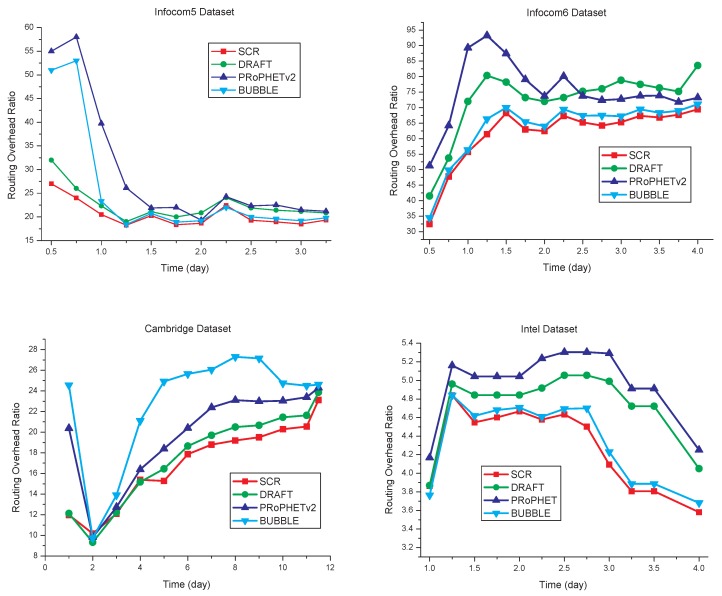
Routing overhead ratio comparisons.

The comparison of routing overhead is shown in Figure 9. Compared with the other three algorithms, SCR has the routing overhead ratio decreased by some extent, as shown in Table 5. Since cluster members have close social relationships in SCR, data transmission has better efficiency than the other three algorithms. In SCR, each node only forwards the copy of the message to nodes that have the destination node as cluster members. Consequently, the total amount of message forwarding is reduced without a negative effect on packet delivery ratio.

### 5.4. Impact of Parameters on Performance

**Figure 10:**
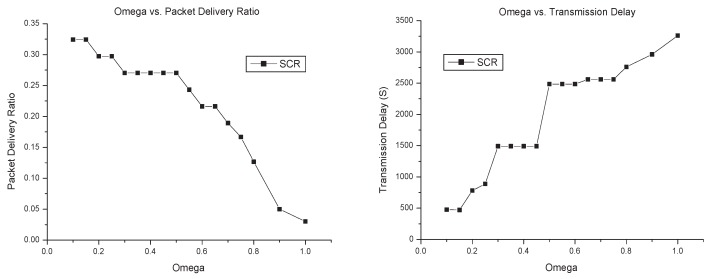

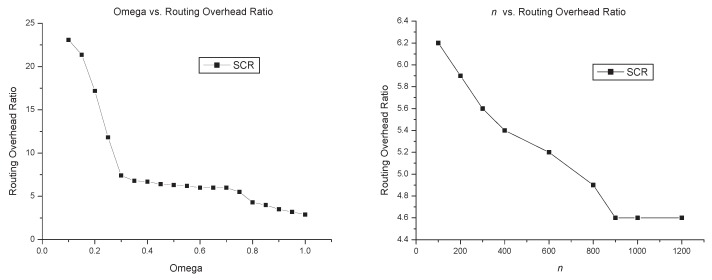
Impact of parameters on SCR performance.

In SCR, there are three parameters: ω, μ and *n*. The parameter ω is the threshold of social relationship strength for cluster members, and to each node, the size of its local cluster will increase with the decreasing of ω. The parameter μ is the referring encounter times for the measurement of social relationship between nodes. In a given condition, the bigger μ has the smaller value of social relationship between two given nodes. For local cluster constructing, ω and μ have a similar effect. On the one hand, with fixed ω, we can change μ to let a node join the cluster. On the other hand, to a given μ, we can change the threshold ω to have a node join the cluster. It is obvious that changing the value of the social relationship has the same effect as changing the threshold of the cluster for cluster constructing. Consequently, we herein only analyse the impact of ω on performance. The parameter *n* is the number of self-decreasing times in the time period *T*, which is for the social relationship to self-decrease to ω, as is shown in Equation (8). Given that the simulation time is three days, the performance of SCR in Infocom5 dataset is shown in Figure 10, where ω is denoted as “Omega”.

As is shown in Figure 10, the smaller ω has the higher packet delivery ratio, the lower transmission delay, and the higher routing overhead ratio. This is consistent with theoretical analysis: the increasing size of the cluster generates more copies of the message forwarded by cluster members, which improves the success of packet transmission and thus decreases the transmission delay, but more copies of the message raises the routing overhead ratio. Considering the tradeoff of packet delivery ratio, transmission delay and routing overhead, we suggest that the parameter ω should be 0.4, which is why we set up ω=0.4 in the above simulation.

From simulation results, the *n* has almost no impact on packet delivery ratio and transmission delay. In our opinion, the *n* has a relationship with the speed of an outdated node dropping from the cluster, but no relationship with the key nodes that can forward messages to destinations successfully. As far as routing overhead is concerned, the smaller *n* leads to more useless nodes staying in the cluster, and more copies of the message forwarded to the network. Therefore, routing overhead ratio increases, as is shown in the lower right part of Figure 10.

## 6. Conclusions

In this paper, we study the effective measurement method for social relationships among nodes in mobile opportunistic networks, and propose a novel social-based clustering and routing scheme. Inspired by the importance of “circles of friends” in the communication networks such as Facebook, Twitter, Wechat and so on, we take a real-life example to show that social relationships among nodes consist of explicit and implicit parts. Explicit social relationships come from direct contact among nodes, and implicit social relationships can be measured through the “circles of friends”. We present the definitions of explicit social relationships (ExSRs) and implicit social relationships (ImSRs), and combine both ExSRs and ImSRs to measure social relationships between nodes. Adaptive weights are given to ExSRs and ImSRs in the measurement of social relationships, which can be adjusted for the contact feature of the nodes, and thus accuracy measurement of social relationships will be achieved. In addition, the distributed computing scheme of common friends is proposed for the calculation of ImSR. Based on effective measurement of social relationships, we propose a novel social-based clustering and routing scheme. Each node selects the nodes with closed social relationships to form a local cluster, and the self-control method is used to keep all cluster members always having close relationships with each other. A cluster-based message forwarding mechanism is designed for opportunistic routing, in which each node only forwards the copy of the message to nodes with the destination node as a member of the local cluster. Simulations have been done, and the results show that the proposed solution outperforms the other two classic routing algorithms, and also shows the effectiveness of the proposed measurement method for social relationships among nodes.

## Figures and Tables

**Table 1:** Characteristics of the four experimental data sets.

Dataset	Infocom5	Infocom6	Cambridge	Intel
Device	iMote	iMote	iMote	iMote
Duration(days)	3.5	4	11.5	4
Number of experimental devices	41	98	52	9
Number of internal contacts iMote	22,459	170,601	10,873	1364

**Table 2:** Simulation parameters of four experimental datasets in ONE.

Dataset	Infocom5	Infocom6	Cambridge	Intel
Number of Nodes	41	98	52	9
Buffer Size	5 M	5 M	5 M	5 M
TTL	60 min	60 min	2 days	0.5 days

**Table 3:** The PDR improvement of SCR compared with the other three algorithms (%).

Algorithms	PRoPHETv2	DRAFT	BUBBLE
Infocom5	18.1	8.5	28.4
Infocom6	8.5	3.7	27.3
Cambridge	4.2	3.4	10.5
Intel	9.5	3.5	14.4

**Table 4:** The TD decrease of SCR compared with the other three algorithms (%).

Algorithms	PRoPHETv2	DRAFT	BUBBLE
Infocom5	17.1	18.2	38.4
Infocom6	29.3	7.6	27.6
Cambridge	5.3	1.4	11.2
Intel	3.3	1.3	5.1

**Table 5:** The ROR decrease of SCR compared with the other three algorithms (%).

Algorithms	PRoPHETv2	DRAFT	BUBBLE
Infocom5	30.7	9.3	19.2
Infocom6	18.5	15.3	3.3
Cambridge	11.2	3.5	15.8
Intel	13.6	9.4	1.5
